# A Statistical Report of Cases at the Hospital of the Sisters of Charity, Buffalo, for One Year, Commencing November First, Eighteen Hundred, and Fifty

**Published:** 1851-12

**Authors:** Charles Ap. A. Bowen

**Affiliations:** Student of Medicine


ART IV. — *A Statistical Report of Cases at the Hospital of the Sisters of Charity, Buffalo, for one year, commencing November first, eighteen hnndred and fifty.* Prepared, from the Hospital Register, by CHARLES AP. A. BOWEN, Student of Medicine.

ADMITTED.

| DISEASES. | November. | December. | January. | February. | March. | April. | May. | June. | July. | August. | September. | October. | Fatals. | Recovered. | Improved. | Totals. |
|---|---|---|---|---|---|---|---|---|---|---|---|---|---|---|---|---|
| I. *Zymotics.* | | | | | | | | | | | | | | | | |
| Cholera Morbus, - - - | | | | | | | | | 2 | | | | 2 | | | 2 |
| Diarrhœa, - - - - - - | 1 | | 3 | 1 | 1 | | | | 6 | 8 | 1 | 1 | | 22 | | 22 |
| Dysentery, - - - - - - | | | 3 | 1 | 1 | | | 1 | 2 | 1 | 1 | 1 | 4 | 7 | | 11 |
| Eczema, - - - - - - | | | | | | | | | 1 | | | | | 1 | | 1 |
| Erythema, - - - - - - | | | | 1 | 1 | | | | | | 1 | | | 3 | | 3 |
| Erysipelas, - - - - - - | | | 1 | | 2 | | 1 | | | 1 | | | | 5 | | 5 |
| Ephemeral Fever, - - - | 4 | | 3 | 1 | 1 | 2 | 1 | 1 | | 1 | 1 | | | 15 | | 15 |
| Intermittent Fever, - - - | 1 | 1 | 1 | 1 | 3 | 4 | 7 | 4 | 5 | 5 | 9 | 5 | 1 | 45 | | 46 |
| Influenza, - - - - - - | | | | | | | | 1 | | | | | | 1 | | 1 |
| Parotitis, - - - - - - | | | 1 | | | | | 1 | | | | | | 2 | | 2 |
| Remittent Fever, - - - | | 2 | 1 | 1 | 5 | 4 | 3 | 10 | | 1 | 5 | 1 | 1 | 32 | | 33 |
| Rubeola, - - - - - - | | | 1 | | | | | | | | | 1 | | 2 | | 2 |
| Scarlatina, - - - - - - | | | 1 | 1 | | | 3 | 1 | | | 1 | 1 | 1 | 7 | | 8 |
| Typhoid Fever, - - - - - | 11 | 14 | 2 | 1 | 5 | 1 | | | 1 | 3 | 8 | 1 | 5 | 41 | 1 | 47 |
| Typhus Fever, - - - - - | | 3 | | | 2 | 1 | | 3 | 3 | 2 | 1 | | 4 | 11 | | 15 |
| Totals, - - - | 17 | 20 | 17 | 8 | 21 | 12 | 15 | 22 | 20 | 22 | 28 | 11 | 18 | 194 | 1 | 213 |
| II. *Sporadics.* | | | | | | | | | | | | | | | | |
| Abscess, - - - - - - | | 2 | | 1 | | | 2 | 1 | | | 1 | | | 5 | 2 | 7 |
| Anasarca, - - - - - - | 1 | | | | | | | | | 1 | | | | 2 | | 2 |
| Carbuncle, - - - - - - | | | | 1 | | | | | | | | | | 1 | | 1 |
| Caries, - - - - - - | | 1 | | 1 | | | 1 | | | | | | | 2 | 1 | 3 |
| Debility, - - - - - - | 1 | | 1 | | 2 | | 2 | | 1 | 2 | 2 | | 1 | 10 | | 11 |
| Dropsy, - - - - - - | | | | | | | 1 | 1 | 1 | | 1 | | 3 | | 1 | 4 |
| Fistula Lachrymalis, - - - | | | | | 1 | | | | | | | | | | 1 | 1 |
| Impetigo, - - - - - - | 1 | | | | | | | | 1 | | 1 | | | 2 | 1 | 3 |
| Herpes, - - - - - - | | | | | | 1 | | | | | | | | 1 | | 1 |
| Pemphigus, - - - - - | | | | 1 | 1 | | | | | | 1 | | | 3 | | 3 |
| Scabies, - - - - - - | | 3 | 3 | 2 | 1 | 1 | | 1 | | | | | | 11 | | 11 |
| Scrofula, - - - - - - | 1 | 1 | | | | | | | | 1 | | | | 1 | 2 | 3 |
| Tinea Capitis, - - - - - | | | | | | | | | 1 | | 1 | | | 2 | | 2 |
| Onychia, - - - - - - | 1 | | | | | | | 1 | | | | 2 | | 4 | | 4 |
| Ulcers, - - - - - - - | | 4 | 1 | 2 | 5 | 5 | 3 | 1 | 1 | 6 | 4 | | | 25 | 7 | 32 |
| Totals, - - - | 5 | 11 | 5 | 8 | 10 | 7 | 9 | 5 | 5 | 10 | 11 | 2 | 4 | 69 | 15 | 88 |
| III. *Nervous System.* | | | | | | | | | | | | | | | | |
| Delirium Tremens, - - - | 1 | | | 1 | 1 | | | | 1 | 1. | | | | 5 | | 5 |
| Epilepsy, - - - - - - | 1 | | | | | 1 | | | | | | 1 | | | 3 | 3 |
| Paralysis, - - - - - - | 1 | 1 | 2 | | | | | | | | | | | 1 | 3 | 4 |
| Neuralgia, - - - - - - | | 1 | | | | 1 | | | | | | | | 1 | 1 | 2 |
| Spinal Irritation, - - - - | | 3 | | 1 | 2 | | | | | | | 2 | | 4 | 4 | 8 |
| Totals, - - - | 3 | 5 | 2 | 2 | 3 | 2 | | | 1 | 1 | | 3 | | 11 | 11 | 22 |
| IV. *Respirative System.* | | | | | | | | | | | | | | | | |
| Catarrh, - - - - - - - | 2 | | | | | | | | | | | | | 2 | | 2 |
| Bronchitis, - - - - - - | 1 | | | | 1 | 2 | 1 | | | 1 | | | 1 | 4 | 1 | 6 |
| Laryngitis, (chronic) - - - | 1 | | | 1 | 1 | | | | | | 1 | 1 | | | 5 | 5 |
| Phthisis Pulmonalis, - - - | 2 | 4 | 2 | 5 | 4 | 1 | 6 | 1 | 3 | 2 | 3 | 1 | 15 | | 19 | 35 |
| Pleuritis, (chronic) - - - | | | | 1 | 1 | | | | | | 1 | | | 1 | 2 | 3 |
| Pneumonitis, - - - - - | 1 | 1 | 1 | 1 | | 2 | 2 | 3 | | | 1 | | 2 | 10 | | 12 |
| Totals, - - - | 7 | 5 | 3 | 8 | 7 | 5 | 9 | 4 | 3 | 3 | 6 | 2 | 18 | 17 | 27 | 62 |

| DISEASES. | November. | December. | January. | February. | March. | April. | May. | June. | July. | August. | September. | October. | Fatal. | Recovered. | Improved. | Totals. |
|---|---|---|---|---|---|---|---|---|---|---|---|---|---|---|---|---|
| V. *Digestive System.* | | | | | | | | | | | | | | | | |
| Abscess, (hepatic) - - - | | | | | | | | | | | 1 | | | | 1 | 1 |
| Cirrhosis, - - - - - - | | | | 1 | | | | | | | | | | | 1 | 1 |
| Dyspepsia, - - - - - - | | | | | 1 | | | 1 | | | | | | 2 | | 2 |
| Hernia, - - - - - - | | 2 | | | | | 1 | | | | | | 1 | 2 | | 3 |
| Hæmorrhoids, - - - - - | | | | 1 | 2 | | | | | | | | | 1 | 2 | 3 |
| Gastritis, - - - - - - | | | | | | | | | 1 | | | | 1 | | | 1 |
| Gastric Irritability, - - - | | | | | 1 | 2 | | 3 | 3 | 1 | | | 1 | 10 | | 10 |
| Icterus, - - - - - - | | | | 1 | | | | | | | | | 1 | | | 1 |
| Totals, - - - | | 2 | | 3 | 4 | 2 | 1 | 4 | 4 | 1 | 1 | | 4 | 15 | 4 | 22 |
| VI. *Circulative System.* | | | | | | | | | | | | | | | | |
| Anæmia, - - - - - - | | | | | | | 2 | | | | | | | 1 | 1 | 2 |
| Hemorrhage, - - - - - | | | | | | | | | | 1 | | | 1 | | | 1 |
| Functional Disease of the Heart, - | | | | | | | 1 | | | | | | | 1 | | 1 |
| Valvular " " " - | 1 | | | | | | | | | | | 1 | | | 2 | 2 |
| Totals, - - - | 1 | | | | | | 3 | | | 1 | | 1 | 1 | 2 | 3 | 6 |
| VII. *Generative System.* | | | | | | | | | | | | | | | | |
| Amenorrhœa, - - - - - | | | | | | | 1 | | 1 | | | | | 2 | 2 | 4 |
| Cancer of Uterus, - - - | | | | 1 | | | | | | | | | 1 | | | 1 |
| Leucorrhœa, - - - - - - | | | | | | | | | 1 | 1 | | | | 2 | | 2 |
| Menorrhagia, - - - - - | | | | | | 1 | | | | | | | | 1 | | 1 |
| Orchitis, - - - - - - | | | | | | | | | 1 | | | | | | 1 | 1 |
| Ovarian Dropsy, - - - - | | | | | | | | | | | | 1 | 1 | | | 1 |
| Paraphimosis, - - - - - | | | | | | 1 | | | | | | | | | 1 | 1 |
| Sarcocele, - - - - - - | | | | 1 | | | | | | | | | | | 1 | 1 |
| Urethritis, - - - - - - | | | | | 1 | | | | | | | | | | 1 | 1 |
| Totals, - - - | | | | 2 | 1 | 2 | 1 | | 3 | 1 | | 2 | 2 | 5 | 6 | 13 |
| VIII. *Locomotive System.* | | | | | | | | | | | | | | | | |
| Coxalgia, - - - - - - | | 1 | 1 | | | | | | | | | | | | 2 | 2 |
| Rheumatism, - - - - - | | | | | 1 | | 2 | 3 | 4 | | | | | 10 | | 10 |
| Synovitis, - - - - - - | | | 1 | | | | | 1 | 1 | | | | | 2 | 1 | 3 |
| Totals, - - - | | 1 | 2 | | 1 | | | | 5 | | | | | 12 | 3 | 15 |
| IX. *Urinative System.* | | | | | | | | | | | | | | | | |
| Albuminaria, - - - - - | | | | | | | | 1 | | | 1 | | | | | 1 |
| Strangury, - - - - - | | | | | | | | | | | 1 | | | | | 2 |
| Totals, - - - | | | | | | | | 1 | | | 2 | | | | | 3 |
| X. *External Causes.* | | | | | | | | | | | | | | | | |
| Accidental, - - - - - - | | | 1 | | 2 | 3 | 3 | 2 | 2 | | 2 | 1 | 2 | 9 | 5 | 16 |
| Amputation of Thigh. - - | | | | | | | | | | | | 1 | | 1 | | 1 |
| " " Leg, - - - | | | | | | | | | 2 | | | | | 2 | | 2 |
| " " Toe, - - - | | | | | | | | | | 1 | | | | 1 | | 1 |
| " at Shoulder Joint, - | | | 1 | | | | | | | | | 1 | | 2 | | 2 |
| " of Penis, - - - | 1 | | | | | | | | | | | | | 1 | | 1 |
| Burns, - - - - - - - | 1 | | | 1 | | | | 1 | 1 | | | | | 4 | | 4 |
| Dislocation of Inferior Extremities, | | 1 | | | | | | | | | | | | 1 | | 1 |
| " of Superior " | | | 1 | | | | | | | | | | | | 1 | 1 |
| Excision of Inferior Maxillary, - | | | 1 | | | | | | | | | | 1 | | | 1 |
| Fracture of Radius, - - - | | | – | | 1 | | | | | | | | | 1 | | 1 |
| " of Radius and Ulna, - | | | | | | | | | | | | | | | | |
| " of Femur, - - - | | 1 | | | 1 | | | | 1 | | | | | 3 | | 3 |
| " of Tibia, - - - | | 1 | | | 1 | | 1 | | | | | | | 4 | | 4 |
| " of Tibia and Tibula, - | | 1 | | | 1 | | 1 | | | | | | 1 | 2 | | 3 |
| " of Patilla, - - - | | | | | 1 | | | | | | | | | 1 | | 1 |
| " of Clavicle, - - - | | | | | | | 1 | | | | | | | 1 | | 1 |
| Wounds contused, - - - | | | 1 | | | 2 | | 1 | | | | | | 4 | | 4 |
| " incised, - - - - | | 1 | | | 1 | 1 | | | | 1 | | | | 3 | | 3 |
| Totals, - - - | 1 | 5 | 6 | 1 | 8 | 6 | 6 | 4 | 6 | 2 | 2 | 3 | 4 | 40 | 6 | 50 |

| DISEASES. | November. | December. | January. | February. | March. | April. | May. | June. | July. | August. | September. | October. | Fatal. | Recovered. | Improved. | Totals. |
|---|---|---|---|---|---|---|---|---|---|---|---|---|---|---|---|---|
| XI. *Opthalmic Diseases.* | | | | | | | | | | | | | | | | |
| Catarrhal Conjunctivitis, - - | 3 | 3 | 4 | 2 | 3 | 4 | 2 | 4 | 4 | 7 | 3 | 1 | | 14 | 25 | 40 |
| Gonorrhæal Opthalmia, - - | | 1 | | | | | | | | | | | | 1 | | 1 |
| Strumous " - - - | | | | | | | | 3 | 1 | | | | | 1 | 2 | 3 |
| Corneitis, - - - - - - | | | | | 2 | | | | 1 | | | | | 2 | 1 | 3 |
| Iritis, - - - - - - | | | | | | | | | | 1 | | | | 1 | | 1 |
| Sclerotitis, - - - - - | | | | | 1 | | | | | | | | | 1 | | 1 |
| Cataract, - - - - - - | | | | 2 | | | | | | | | | | 1 | 1 | 2 |
| Totals, - - - | 3 | 4 | 4 | 4 | 6 | 4 | 2 | 3 | 6 | 8 | 3 | 1 | | 21 | 29 | 50 |
| XII. *Venereal Diseases.* | | | | | | | | | | | | | | | | |
| Gonorrhœa, - - - - - - | | 1 | | | | | | | | 1 | 1 | | | 1 | 2 | 3 |
| Syphilis Primary, - - - | | | | | | 1 | 2 | | | 2 | 1 | | | 3 | 3 | 6 |
| " Secondary, - - - | | 2 | 2 | | 1 | | 2 | | | | | | | 1 | 6 | 7 |
| " Tertiary, - - - | 1 | | | | | | 1 | 2 | 1 | 2 | 1 | 1 | | 1 | 8 | 9 |
| Totals, - - - | 1 | 3 | 2 | | 1 | 1 | 5 | 2 | 1 | 5 | 3 | 1 | | 6 | 19 | 25 |
| XIII. *Miscellaneous.* | | | | | | | | | | | | | | | | |
| Enlarged Spleen, - - - - | | | | | | 2 | | | | | | | 1 | | 1 | 2 |
| " Gland of Neck, - - | | 1 | | | | | | | | | | | | 1 | | 1 |
| Frost Bites, - - - - - - | | | 1 | | 1 | | | | | | | | | 2 | | 2 |
| Pains in limbs, back, &c., - - | | 2 | | | 1 | | | | | | | | | 3 | | 3 |
| Totals, - - - | | 3 | 1 | | 2 | 2 | | | | | | | 1 | 6 | 1 | 8 |
| XIV. *Diseases not Specified.* | 1 | 2 | 5 | 1 | 5 | 1 | | 1 | 3 | 10 | 6 | 8 | | | | 43 |

### RECAPITULATION.

| | | | | | | | | | | | | | | | | |
|---|---|---|---|---|---|---|---|---|---|---|---|---|---|---|---|---|
| 1. Zymotics, - - - - - | 17 | 20 | 17 | 8 | 21 | 12 | 15 | 22 | 20 | 22 | 28 | 11 | 18 | 194 | 1 | 213 |
| 2. Sporadics, - - - - - | 5 | 11 | 5 | 8 | 10 | 7 | 9 | 5 | 5 | 10 | 11 | 2 | 4 | 69 | 15 | 88 |
| 3. Nervous System, - - - | 3 | 5 | 2 | 2 | 3 | 2 | | | 1 | 1 | | 3 | | 11 | 11 | 22 |
| 4. Sespirative System, - - | 7 | 5 | 3 | 8 | 7 | 5 | 9 | 4 | 3 | 3 | 6 | 2 | 18 | 17 | 27 | 62 |
| 5. Digestive System, - - - | | 2 | | 2 | 3 | 4 | 1 | 4 | 4 | 1 | 1 | 1 | 3 | 15 | 4 | 22 |
| 6. Circulative System, - - | 1 | | | | | | 3 | | | 1 | | 1 | 1 | 2 | 3 | 6 |
| 7. Generative System, - - - | | | 2 | 2 | 1 | 2 | 1 | | 3 | | 1 | 1 | 2 | 5 | 6 | 13 |
| 8. Locomotive System, - - | | 1 | 2 | | 1 | | 2 | 4 | 5 | | | | | 12 | 3 | 5 |
| 9. Urinative System, - - - | | | | | | | | 1 | | | 2 | | 1 | 2 | | 3 |
| 10. External Causes, - - - | 2 | 5 | 6 | 1 | 8 | 6 | 6 | 4 | 5 | 2 | 2 | 3 | 4 | 40 | 6 | 50 |
| 11. Ophthalmic Diseases, - - | 3 | 4 | 4 | 4 | 6 | 4 | 2 | 6 | 6 | 8 | 3 | 1 | | 21 | 29 | 50 |
| 12. Venereal Diseases, - - | 1 | 3 | 2 | | 1 | 1 | 5 | 2 | 1 | 5 | 3 | 1 | | 6 | 19 | 25 |
| 13. Miscellaneous, - - - - | | 3 | 1 | | 2 | 2 | | | | | | | 1 | 6 | 1 | 8 |
| 14. Diseases not Specified, - - | 1 | 2 | 10 | 4 | 6 | 1 | | 1 | 3 | 14 | 6 | 8 | | | | 56 |
| Totals, - - - | 40 | 61 | 54 | 39 | 69 | 46 | 52 | 53 | 56 | 67 | 63 | 34 | 52 | 400 | 125 | 623 |

